# Refining MediQuit: an iterative, participatory approach to shared decision-making in deprescribing

**DOI:** 10.1186/s43058-026-01022-w

**Published:** 2026-06-17

**Authors:** Nele Kornder, Sarah Burkhardt, Konrad Hierasimowicz, Annika Viniol, Norbert Donner-Banzhoff

**Affiliations:** Department of Primary Care, Marburg University, Karl-von-Frisch-Straße 4, 35043 Marburg, Germany

**Keywords:** Deprescribing, Shared decision making, Primary care, Polypharmacy, Digital decision aid, Implementation science, Patient-centered care, Complex interventions

## Abstract

**Background:**

Polypharmacy and potentially inappropriate medications are highly prevalent in primary care and are associated with adverse drug events, reduced adherence, and diminished quality of life. Deprescribing is a key strategy to address these challenges, but its implementation is complex, particularly when long-term preventive medications are involved and decisions are preference-sensitive. Digital tools may support shared decision making in deprescribing, yet many existing tools lack clear implementation orientation. This study describes the iterative refinement of arribaMediQuit, a digital deprescribing tool for primary care, with the aim of improving usability, ethical robustness, and implementation potential.

**Methods:**

The refinement followed an iterative, participatory development process informed by the Medical Research Council framework for complex interventions and the International Patient Decision Aid Standards. Multiple stakeholder groups were involved, including general practitioners, researchers in health services and pharmacology, members of a patient advisory board, experts in medical ethics, and the original developers of the tool. Regular expert meetings were used to review content, terminology, visual design, decision logic, and deprescribing strategies. Feedback was continuously integrated into successive versions of the tool. The study focused on qualitative refinement rather than outcome evaluation.

**Results:**

Key refinements included the development of a dynamic medication database and a structured categorization of medications into three categories (symptomatic, intermediate, and preventive medications) with tailored decision processes. A new linear decision model was introduced for preventive medications to better reflect value-sensitive trade-offs under uncertainty. Terminology and visual elements were revised to align with everyday clinical language and to enhance patient comprehensibility. Ethical considerations, including the communication of benefits, harms, and withdrawal symptoms, were explicitly addressed. Stakeholders also identified potential future uses of the tool, such as educational applications and integration with other deprescribing or medication review tools.

**Conclusions:**

The iterative, theory-informed refinement of arribaMediQuit illustrates how shared decision making principles can be operationalized in a deprescribing tool designed for routine primary care. By integrating technical guidance with value-sensitive deliberation and implementation considerations, the tool shows promise for supporting ethically grounded and feasible deprescribing. Future studies will evaluate feasibility, acceptability, and use in routine practice.

## Contributions to the literature

- This paper describes the iterative, stakeholder-informed refinement of a digital deprescribing tool, illustrating how complex interventions can be further developed with early attention to implementation.
- It shows how technical problem-solving elements (e.g. medication review and tapering guidance) can be combined with value-sensitive shared decision making within routine primary care consultations.
- The study highlights that future implementation may depend not only on tool content, but also on explicitly clarifying when and how decision aids are intended to be used in everyday clinical workflows.
- By integrating established frameworks (Medical Research Council framework, International Patient Decision Aid Standards, and implementation-oriented thinking), the paper offers transferable insights for the development and testing of similar decision aids in primary care.

## Introduction

### Background

Polypharmacy represents a major challenge in primary care, as it is closely linked to adverse drug events [1, 2] reduced treatment adherence [3] and diminished quality of life among older adults [4]. Deprescribing—defined as the process of reducing or discontinuing inappropriate medications [5] - has therefore become a central strategy to improve care in this population. At the same time, deprescribing is not without risks. Potential harms include adverse drug withdrawal reactions, recurrence of underlying conditions, reversal of drug–drug interactions, and strain on the doctor–patient relationship [5, 6]. These concerns can lead to uncertainty and reluctance among both clinicians and patients, contributing to the continuation of potentially inappropriate medications [7].

Deprescribing decisions are inherently complex, as they require weighing two competing perspectives: the potential benefits of continuing treatment for disease control and prevention, and the potential harms of ongoing medication use, including adverse effects and treatment burden [8].

Addressing these challenges requires more than simply stopping medications. While unilateral discontinuation by clinicians may appear efficient, it risks overlooking patient concerns, preferences, and contextual factors that are central to safe and acceptable deprescribing. Instead, evidence suggests that potential risks can be minimized through a systematic, patient-centered process that includes careful planning, and—depending on the medication—tapering and monitoring [9]. An important requirement for informed decision-making by both clinicians and patients is knowledge of the effect sizes of benefits and harms of pharmacotherapy. The indication for a medication arises from weighing these two dimensions, which in practice are only known as probabilities derived from clinical studies. Two large systematic reviews [10, 11] have demonstrated that clinicians and patients rarely have accurate expectations regarding the benefits and harms of medical interventions, including drug treatments. Inaccurate estimates more often involve overestimation of benefits and underestimation of harms, which may contribute to overtreatment. This misperception highlights the importance of knowledge transfer (providing of information etc.) for deprescribing decisions, before patients’ values and preferences are addressed. The latter is the hallmark of shared decision making (SDM) which provides an essential framework for the desprescribing task. However, as a recent scoping review points out, existing interventions differ widely in their operationalization of SDM, and its impact on patient outcomes has seldom been systematically assessed [12]

However, many existing deprescribing tools remain insufficiently adapted to routine practice, reflecting broader implementation challenges related to workflow integration and healthcare system contexts [13]. Ethical considerations and patients’ individual life goals are frequently neglected, and it is often unclear whether the tools primarily function as technical aids (e.g., tapering guidance) or as instruments to facilitate meaningful dialogue between clinicians and patients. This highlights a gap in available tools and strategies: They not only have to be evidence-based and patient-centered but also feasible and usable in real-world primary care.

### MediQuit: a digital consultation tool for deprescribing

To address these challenges, the digital consultation tool arribaMediQuit (hereafter referred as MediQuit) has been developed. It is designed to be used by primary care providers during face-to-face consultations in routine practice. MediQuit is based on arriba (https://arriba-hausarzt.de/), a digital library of participatory decision aids. Each module addresses a specific clinical decision (e.g., cardiovascular prevention or type 2 diabetes management) and integrates evidence from epidemiological, diagnostic, and clinical studies. The potential effects of therapeutic and preventive interventions are dynamically visualized using smileys, diagrams, and decision scales, enabling individualized, evidence-based decisions to be made collaboratively during the consultation.

MediQuit guides primary care providers through the complex deprescribing process by supporting three main steps: (1) identifying a potentially inappropriate medication, (2) engaging in a structured consultation with the patient to enable SDM, and (3) providing practical guidance to support implementation of the agreed plan in patients’ daily lives, including structured follow-up and ongoing monitoring.

**Fig. 1:**
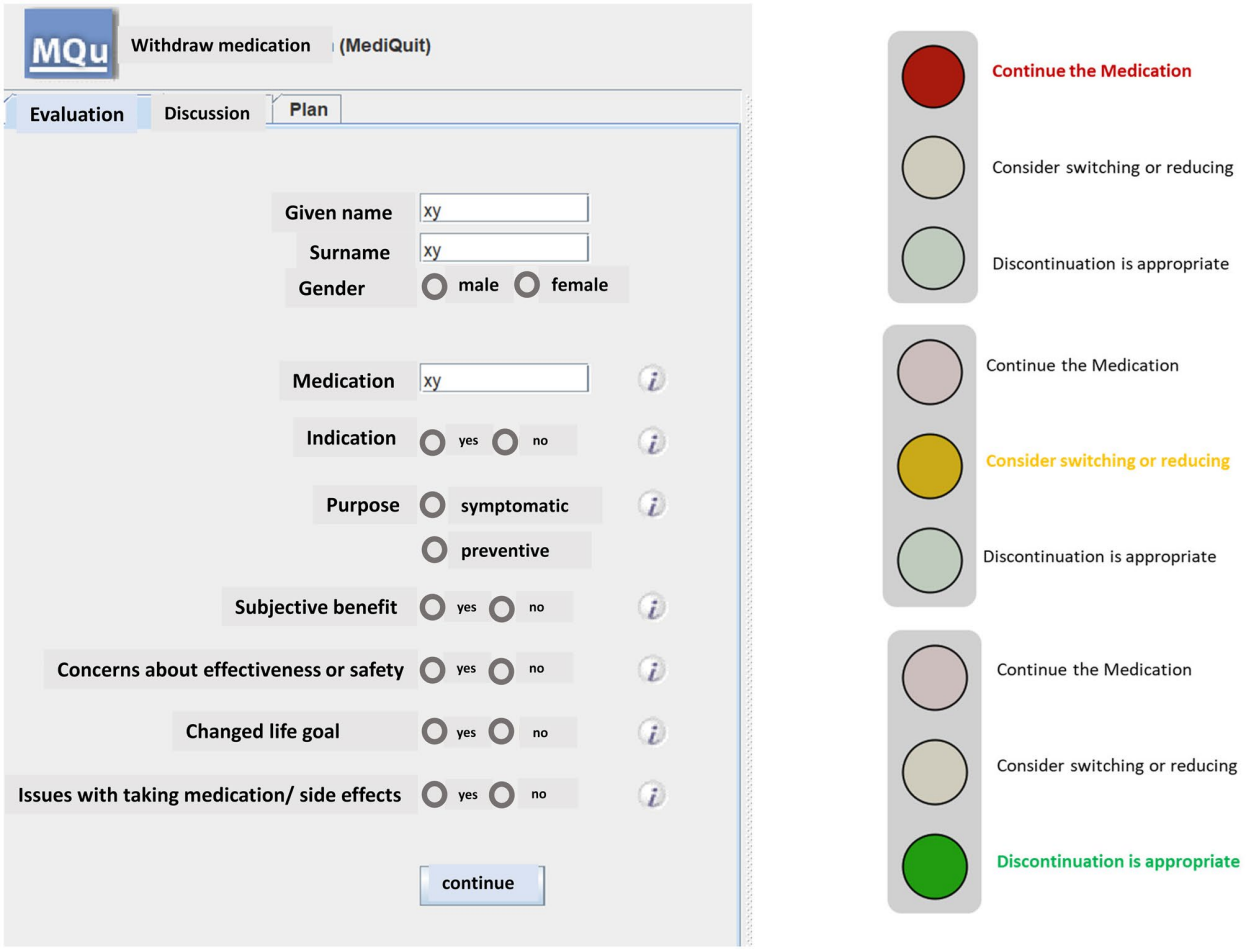
Screenshot of the original arribaMediQuit tool with a traffic-light system used to present recommendations (translated from German by the authors)

In its former version, the tool was structured as an interactive, screen-based consultation aid. On the left side of the screen, the primary care provider entered the selected medication and relevant patient-specific information, and then worked through a series of steps. Based on these entries, the tool generated communication prompts and deprescribing suggestions, which were displayed on the right side of the screen using a traffic-light logic (red–yellow–green) to indicate the overall direction of the recommendation. These visual and textual elements were intended to provide general guidance and did not contain specific information for individual medications or medication groups. They were meant to support, rather than predetermine, the ensuing dialogue with the patient (see Fig. 1).

In a pilot study, MediQuit was well received by both primary care physicians and patients, who valued its stepwise approach to SDM [14]. At the same time, several areas for improvement were identified. Primary care physicians expressed the need for a more intuitive design, more concrete support for SDM, and specific tapering suggestions for selected medication groups [14].

These findings provided the basis for the current further development, which explicitly integrates theoretical frameworks (Medical Research Council (MRC) framework for complex interventions [15] and the International Patient Decision Aid Standards (IPDAS) criteria [16]) to optimize feasibility, usability, and potential for successful implementation in routine primary care.

### Aim

The aim of this study was to iteratively and participatively revise an existing digital deprescribing tool (MediQuit) for use in general practice. Specifically, the study sought to:

- Involve key stakeholders—such as, primary care providers, patients, and researchers—in a co-creative process to refine the tool’s content, structure, and usability.
- Identify and implement modifications that enhance the tool’s practical relevance, SDM, and address conceptual and ethical challenges encountered in deprescribing consultations.
- Generate insights and practical considerations that may inform the future development, implementation, and evaluation of digital decision aids for deprescribing and related primary care contexts.

## Methods

**Fig. 2:**
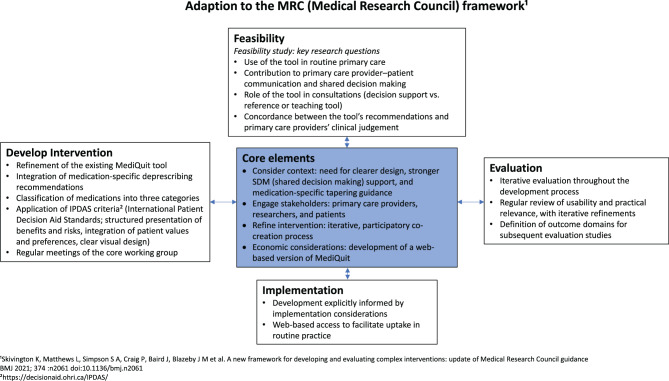
Development of arribaMediQuit following the MRC framework and incorporating core IPDAS criteria

MediQuit was subsequently refined through an iterative, participatory co-creation process guided by the MRC framework for complex interventions [15]. Situated within the development phase of the MRC framework, this study focused on systematically optimizing the tool’s design, content, and usability prior to formal feasibility and evaluation testing (see Fig. 2). Building on findings from an initial pilot study of the original tool, we conducted a structured, multi-stage development process that integrated input from a wide range of stakeholders.

During the iterative development of MediQuit, key principles from the IPDAS [16] were applied to guide the design and evaluation of the tool. Specifically, these principles informed the structured presentation of evidence on benefits and risks, the integration of patient preferences and values into the decision-making process, and the design of clear visual and interactive elements to support SDM.

**Fig. 3:**
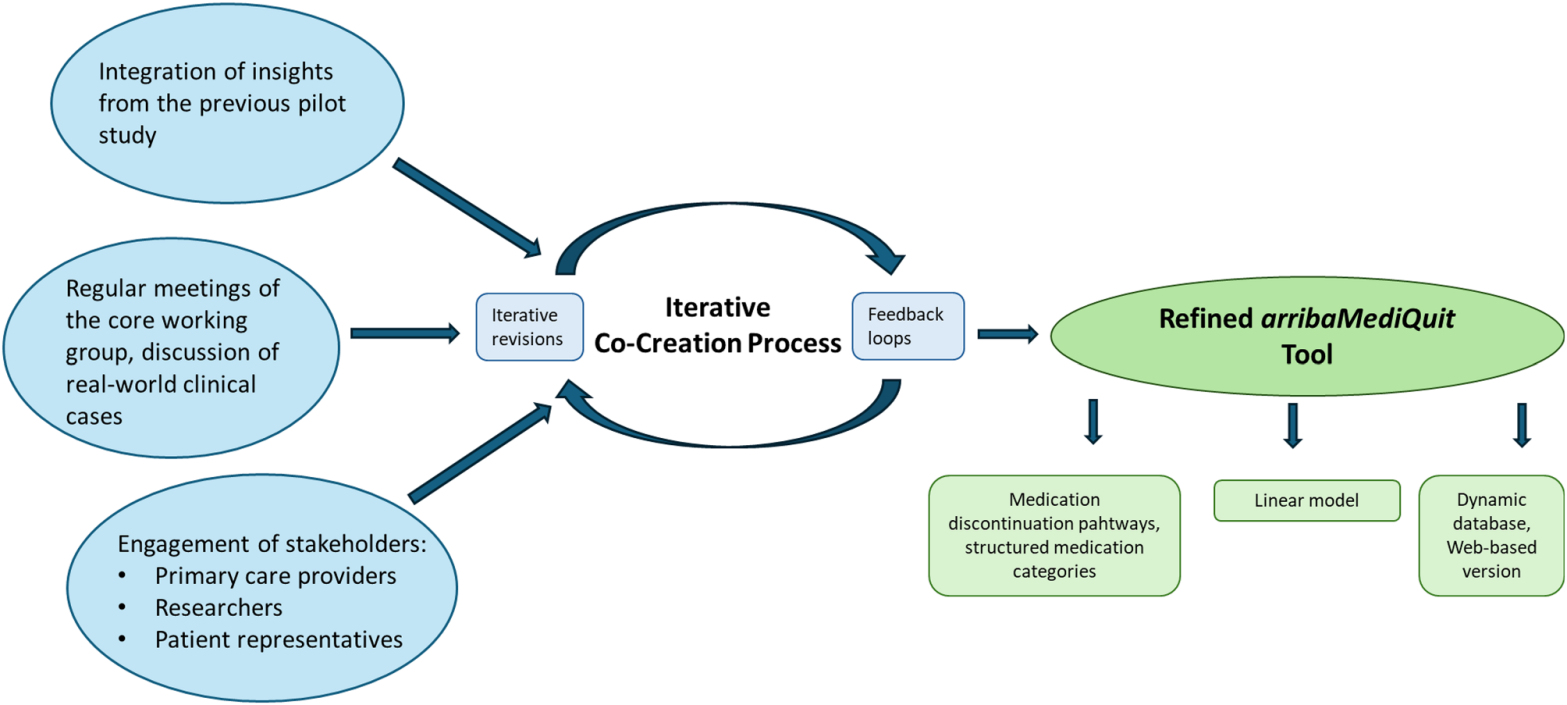
Refinement process of arriba MediQuit. This figure illustrates the iterative and participatory refinement of arribaMediquit. The process was informed by insights from a previous pilot study and involved regular meetings of the core working group. Through iterative revisions and feedback loops, stakeholder input and practical considerations were continuously integrated

Our iterative co-creation process incorporated stakeholder engagement from primary care providers, patients, researchers, and the original developers. The overall refinement process and its iterative stages are illustrated in Fig. 3.

The specific stages of the development process are described in detail below.

### Phase I – starting point and preliminary findings

The core working group (NK, SB, NDB, AV, KH) initiated the revision process based on findings from a previously published pilot study of the original MediQuit tool [14].

The core team consisted of academic primary care physicians and a researcher with a background in informatics who was responsible for the technical implementation of the tool. NDB, NK, AV and KH had previously been involved in the design and pilot testing of other SDM tools (e.g., arriba-PPI [17], arriba-ExPa [18]), providing valuable methodological and practical insights that informed the refinement process.

While no formal systematic review was conducted, the team reviewed key literature on strategies for deprescribing and their implementation [19–21]. Insights from previous arriba projects [17, 18, 22] provided both conceptual and empirical evidence regarding the challenges and success factors of implementing SDM tools in general practice.

For the deprescribing recommendations, the process was guided by relevant clinical guidelines and existing deprescribing frameworks, including COFRAIL [23] and national and international deprescribing guidance like medstopper [24]. In addition, commonly used pharmacotherapy resources (e.g. the Gelbe Liste, a pharmaceutical directory providing information on medicinal products approved in Germany, such as active substances, indications, dosage forms, and dosage [25]) were consulted to ensure that dosing, indications, and tapering suggestions were consistent with routine deprescribing practice. Together, these sources ensured that the resulting tool is both evidence-informed and clinically feasible for use in routine general practice.

### Phase II – participatory and iterative redevelopment process

The refinement followed an iterative and participatory approach. A broad range of stakeholders was involved throughout the process, including (1) primary care physicians with practical experience in multimorbidity and polypharmacy (for example, the project was presented to members of the arriba cooperative, a non-profit association of primary care providers that promotes evidence-based information and the participatory involvement of patients in preventive, diagnostic, and therapeutic decision-making), (2) researchers in health services and pharmacology, (3) members of a patient advisory board, (4) external experts in deprescribing research and digital tool development, (5) the original developers of MediQuit, and (6) representatives with expertise in medical ethics. In this context the project was presented, among others, to the German Deprescribing Network, a collaborative network of researchers from general practice, geriatrics, clinical pharmacology, pharmacy, and other disciplines. One of the aims of this network is the exchange of methodologies for research projects related to deprescribing, as well as international collaboration with other deprescribing networks (https://deprescribing.de/). Depending on the stakeholder group and format, between 1 and 15 participants were involved in the respective activities. This included, for example, one medical ethicist, a patient advisory board comprising seven members, smaller expert meetings involving 2–6 academic primary care physicians and researchers, and larger multiprofessional groups of up to 15 participants (e.g. within the German Deprescribing Network).

The iterative nature of the process was emphasized through regular team meetings, during which feedback from prior consultations was reviewed, discussed, and systematically integrated into subsequent versions of the tool. Stakeholders were consulted as soon as the core working group reached a stage of revision that required external input or validation.

The insights gained from this process informed iterative refinements of the tool.

### Phase III – consolidation and final version

All discussion points and stakeholder contributions were systematically documented and reviewed by the core working group. This led to the consolidation of a revised version of MediQuit.

In parallel, key feasibility and implementation questions, as well as outcome domains for the upcoming study, were defined – with a particular focus on usability in routine consultations, acceptability for both primary care providers and patients, integration into existing workflows, and anticipated barriers and facilitators to use.

The final version is now being prepared for feasibility testing in routine primary care. Ethical approval for the feasibility study has been obtained (reference no. 25–315). The overarching goal is to enable effective future implementation of the tool in everyday practice.

To improve clarity and readability of the manuscript, AI-assisted language editing (ChatGPT, OpenAI) was used. All content was reviewed and approved by the authors.

## Results

The core working group met regularly (approximately every two weeks over a period of 16 month) to iteratively discuss and refine the tool. These meetings allowed the team to continuously integrate feedback from stakeholders and to incorporate practical considerations arising from the members’ parallel work as primary care physicians in routine primary care.

### General design and technical adaptations

In Phase I, the existing tool was refined through a set of overarching adjustments at the level of wording, structure, and visual presentation, with the aim of improving usability and implementation potential. Wording and labeling of individual items were revised to enhance clarity and better reflect everyday clinical language. For example, the term *“indication”* was changed to *“therapy goal”* to more clearly convey the purpose of a medication in patient care and support meaningful discussions. Attention was also paid to creating an intuitive interface, reducing ambiguity, and aligning terminology with other arriba decision aids.

Second, and importantly from an implementation perspective, MediQuit was migrated from a locally installed Java-based application to a web-based version. Previous experiences with decision aids of the arriba library had shown that dependence on specific software (such as a Java runtime environment) and the need to download and install a program can constitute a substantial barrier to uptake in busy primary care settings. By providing a browser-based solution, the revised tool is now accessible from standard practice computers across different operating systems without additional installation steps, thereby lowering technical entry barriers and supporting smoother integration into routine workflows.

### Medication grouping and pathway development

Early feedback from an expert panel of primary care providers from the arriba cooperative—a non-profit association that promotes evidence-based information and patient participation in medical decision-making—highlighted the need for guidance on specific medication groups commonly encountered in primary care, as well as typical examples of medications considered for deprescribing.

This feedback closely aligned with findings from the initial pilot study, which had similarly indicated a demand for more concrete, medication-specific recommendations. This included the development of more concrete, medication-specific deprescribing recommendations, such as guidance on tapering strategies, monitoring requirements, and follow-up considerations for selected medication groups. The panel suggested a range of drugs, including analgesics, statins, thyroid medications and antidepressants.

A key goal identified from the outset was the development of a dynamic medication database that could be continuously expanded. Given the heterogeneity of medications and their diverse mechanisms of action, the core expert team recognized the need for a structured classification system. This led to the definition of three distinct medication categories comprising drugs with similar pharmacological and clinical characteristics. These categories allow a degree of standardization of outputs while still accommodating clinically relevant differences. Each category is linked to a specific pathway within the tool, guiding clinicians and patients through a structured decision-making process and ensuring that deprescribing steps are tailored to the characteristics of the respective medication category.

Category I – Symptomatic medications: These drugs primarily address symptoms without significantly affecting the underlying disease. Deprescribing effects are typically immediate and noticeable, allowing patients to directly perceive improvements or worsening of symptoms. Examples include analgesics, anticonvulsants used for pain therapy, and antianginal medications.

Category II – Intermediate medications: These medications target specific conditions, but their effects are neither purely symptomatic nor purely preventive. Deprescribing may lead to perceptible effects, though these are usually not immediate. Typical examples include levothyroxine for hypothyroidism, antidepressants, and antihypertensive drugs. Monitoring may involve objective data (e.g. laboratory measurements, vital signs) and/or subjective data (e.g., patient-reported outcomes), depending on the medication and clinical context, to ensure safe discontinuation.

Category III – Preventive medications These drugs are primarily used for secondary prevention of disease progression or for primary prevention. Statins are a typical example of this group. Deprescribing effects are generally long-term and random, making monitoring more challenging. Surrogate measures are available but capture only small part of the association between treatment and events. Decisions in this group are particularly value sensitive -, as they often involve weighing long-term, uncertain benefits against current treatment burden and potential harms. Individual life goals, patient preferences, and ethical considerations therefore play a central role when discussing deprescribing in this category. For this group, monitoring is limited and immediate clinical changes are rarely observed. Therefore, follow-up primarily involves periodic clinical reassessment of the patient’s overall health status and reconsideration of treatment decisions over time, rather than routine laboratory monitoring.

The categorization was iteratively developed and validated within the core team through repeated discussions of individual medication examples. These discussions helped ensure that the three-group scheme was conceptually coherent and applicable across a broad range of drugs commonly encountered in general practice.

While most medications can be clearly assigned to one of these three groups, some drugs may occupy different categories depending on the primary treatment goal. Beta-blockers, for example, can be used as symptomatic agents in certain forms of tachycardia (Group I), but also as Group II medications in conditions such as hypertension, where symptomatic and preventive effects are intertwined. Lastly, they can have preventive effects and thus belong to Group III when they are, for example, used for secondary prevention in vascular disease. In most instances the drug determines the category I to III. If, however, one particular drug belongs to more than one, primary care providers assign the patient’s medication based on the individual therapeutic goal. This flexibility allows MediQuit to reflect the clinical reality that deprescribing decisions are determined not only by drug class, but also by the specific therapy goal, context, and treatment intent for each patient.

By structuring MediQuit around these three groups, the tool provides tailored guidance for the deprescribing process while maintaining flexibility. For each category, a category-specific sequence of decision and information steps was developed, guiding users through the tool—from entering medication- and patient-related information, through clinical assessment and technical problem-solving steps (e.g. indication review, monitoring requirements, tapering considerations), to value-sensitive deliberation and a structured deprescribing recommendation. Considerable attention was paid to determining for which medications and which clinical scenarios it would be meaningful to incorporate discussions of individual life goals, patient preferences, and ethical considerations. These deliberations directly informed the creation of the linear model for Group III medications, which is described in detail in the following section.

New medications can be integrated into the existing database by assigning them to the appropriate category and linking them to the corresponding pathway, ensuring that the tool remains adaptable and clinically relevant across different therapeutic areas.

### Development of a linear model for patient-centered deprescribing

To better integrate patient preferences and life goals into the deprescribing process for preventive medications in category III we developed a linear model that maps key factors influencing decision-making. These factors include age, morbidity, frailty, accepted treatment burden and preference for future prevention of organ damage.

To support structured decision making, each factor is assessed on a scale from 0 to 10. Morbidity and frailty are evaluated by the primary care provider based on clinical judgement, with frailty informed by the Clinical Frailty Scale [26] and its German adaption [27]. In contrast, accepted treatment burden and preference for future prevention of organ damage are based on the patient’s subjective assessment and are explored during the consultation.

The factor “preference for future prevention of organ damage” is particularly relevant for preventive medications, as potential benefits often occur only over a longer time horizon. It is intended to capture how important such future benefits are from the patient’s perspective, taking into account the expected time to benefit.

The new linear model was partly informed by the arribaDiabetes tool which, although developed for a different clinical context, operationalizes core principles already required by clinical guidelines, such as considering dose reduction or discontinuation of medication in light of glycaemic control, comorbidity, and treatment burden. In this broader sense, arribaDiabetes addresses decisions that can be understood as deprescribing and integrates patient life goals alongside clinical parameters into these choices, thereby supporting the content validity of both modules [22].

**Fig. 4:**
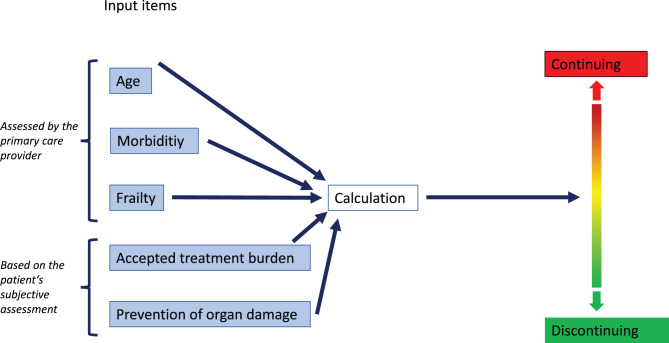
Underlying principle of the “new” linear model. The primary care provider assigns a score to each input item. Based on these scores, it is calculated where the recommendation falls on the spectrum between discontinuing and continuing the medication. All variables represent patient-related factors. Clinical variables (e.g. age, morbidity, frailty) are assessed by the primary care provider, while treatment burden and prevention of organ damage are based on the patient’s subjective assessment in relation to the specific medication under discussion. Double click the image to add any comment

**Fig. 5:**
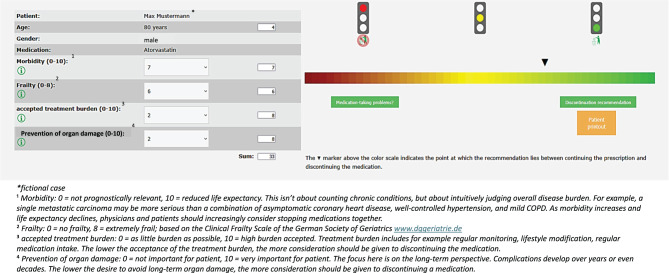
The “new” linear model for preventive medication (group III)

For preventive medications in category III, a key modification compared to earlier versions of the tool was the move from a simple red–yellow–green traffic light system to a continuous color gradient, on which an arrow indicates the position reached within the patient–primary care provider conversation (see Figs. 4 and 5). This visualization underscores that decisions in these domains are rarely clear-cut or binary. Instead, they often represent subtle, highly individual tendencies rather than fixed categories, reflecting nuanced trade-offs between potential long-term benefits and current treatment burden.

For the underlying scoring system, each category was initially assigned a point range, with each point value linked to a specific position of the arrow on the continuous color scale. The core team then applied the preliminary scoring to real-world case examples from their own practice, iteratively adjusting the model to better reflect clinical judgement.

The numerical scale (0–10) is not intended as a precise quantitative measure, but rather as a subjective assessment to support reflection and discussion of individual values within the SDM process.

The linear model was presented to the department’s patient advisory board to assess comprehensibility and to gather feedback on terminology, clarity, and practical usability. Overall, the model was perceived as meaningful and helpful for structuring deprescribing decisions. Input from the patient representatives was incorporated into the model, helping to refine the categories and ensure that the tool communicates complex information in a patient-friendly manner. During these discussions, broader questions arose about how to communicate potential withdrawal symptoms and other risks in a balanced, patient-centered manner, which then informed further refinements to the tool (as described in detail in the following section).

In addition, stakeholders perceived the continuous color gradient as a more nuanced representation compared to the previous traffic light system. However, its impact on usability and decision-making in routine practice will be further evaluated in the feasibility study.

In discussion with a medical ethicist, the linear model was also examined from an ethical perspective. A key question was whether it is ethically acceptable to aggregate complex, value-laden dimensions—such as life goals, treatment burden and morbidity—into a single score. While this reduction was acknowledged as inherently imperfect, the group agreed that some form of structured aggregation is unavoidable to support clinical deliberation. Importantly, the score is intended as a heuristic to prompt reflection and dialogue rather than as a prescriptive decision rule. It was also discussed that conversations about life goals within the model may benefit from explicitly addressing whether patients perceive their lives as having been fulfilling, as this can provide important context for deprescribing decisions.

### Withdrawal symptoms: how much information is helpful?

Within the core working group, one central issue that emerged during the development of MediQuit concerned how extensively potential withdrawal symptoms should be addressed in the consultation. Analogous to discussions about adverse effects when initiating medications, concerns were raised about whether detailed information on discontinuation symptoms might discourage patients from discontinuation of a drug or even contribute to nocebo effects. This topic was subsequently taken up and further explored with the institute’s patient advisory board, who emphasized the importance of transparent information while also acknowledging that patients differ in how much detail they wish to receive. The group agreed that information on withdrawal symptoms should be available within the tool but tailored to the clinical context. In the current version of arribaMediQuit, withdrawal symptoms are therefore treated as an optional discussion element that can be addressed according to the patient’s preferences, information needs, and perceived coping resources. Relevant information is accessible to the physician within a dedicated information box and can, where appropriate, be selectively added by the clinician to the patient printout. This approach aims to strike a balance between respecting autonomy and providing sufficient information on the one hand, and avoiding unnecessary anxiety or unintended nocebo effects on the other hand.

### Suggestions for future use and integration

Stakeholders also identified several ideas for future use of MediQuit. One suggestion was to employ the tool as an educational resource—for medical students and physicians in training—to teach structured deprescribing, risk communication, and SDM. In addition, participants proposed integrating the tool with existing deprescribing instruments or preceding it with a brief medication check to streamline workflows.

### Next step: feasibility study

Building on the insights gained during the iterative redevelopment of MediQuit, a mixed-methods feasibility study is planned to assess the tool’s integration into routine primary care and develop effective implementation strategies. The study will involve ten primary care physicians from middle and northern Hesse, each discussing deprescribing issues with three to four patients over six weeks. Participating patients are adults who regularly take medications included in MediQuit and are able to engage in SDM consultations.

The study’s research questions—covering patterns of use, perceived utility in consultations, and contextual factors affecting adoption—are directly derived from findings during the refinement process. In particular, targeted questions addressing implementation challenges were already explored and further developed during this phase. Quantitative measures include frequency and duration of tool use, targeted medication groups, and use of key functions such as visualizations and deprescribing plans. Qualitative assessments focus on primary care physicians’ experiences with the tool, its role in supporting SDM versus serving as a reference or educational resource, and practical barriers and facilitators to integration. Semi-structured interviews with participating primary care providers will provide deeper insights into these implementation-relevant factors.

This feasibility study is intended to provide preliminary evidence on the acceptability, usability, and practical integration of MediQuit in primary care, guiding further refinement and informing the design of larger-scale implementation and evaluation studies.

## Discussion

### Conceptual foundations for shared decision making in deprescribing

During expert discussions, a key question concerned the point in the clinical encounter at which the tool should be used. This reflects a common challenge in SDM implementation—whether tools are intended primarily for structured guidance (e.g. how to taper medications) or to facilitate dialogue, reflection, and alignment of decisions with patients’ values.

This tension can be understood as a distinction between problem solving and decision making in clinical care. While problem solving focuses on technical tasks with a largely “correct” solution, decision making involves value-sensitive choices between reasonable alternatives, where no single “right” answer exists [28, 29]. Patients generally wish to be more involved in decision making than in problem solving, highlighting the need for tools that not only support technical aspects of deprescribing but also enable deliberation and value clarification [30]. Recent research further suggests that patients differ in their attitudes towards medications and their preferred level of involvement in deprescribing decisions [31, 32]. While MediQuit does not explicitly classify patients into predefined types, the tool is designed to accommodate this variability by supporting flexible, patient-centred discussions that integrate individual preferences, values, and desired levels of involvement.

Taken together, these considerations highlight the importance of supporting both technical guidance and value-sensitive deliberation when developing deprescribing tools. MediQuit was designed with this in mind, combining structured support for clinical decision making with space for shared deliberation in routine care.

### Balancing transparency and patient-centered risk communication

A further ethical and practical consideration concerns the communication of potential withdrawal symptoms. As reflected in discussions with the patient advisory board during the refinement process, patients differ considerably in how much information they wish to receive about potential discontinuation effects. To balance transparency with patient-centered care, MediQuit therefore treats withdrawal symptoms as an optional discussion element that can be addressed according to individual patient preferences, coping capacity, and desired level of detail. In this way, the tool supports informed and value-sensitive decision making while reducing the risk of unnecessary anxiety or nocebo effects [33–35].

This finding highlights the importance of flexible, patient-centered approaches to risk communication in deprescribing, allowing clinicians to tailor information to individual needs rather than applying a one-size-fits-all strategy. Furthermore, recent work by Jagosh et al. highlights how “prescribing inertia” in end-of-life care is shaped by the symbolic meaning of medications, clinician reluctance, and the need for ethically sensitive conversations [36]. MediQuit aims to counter such inertia by structuring value-based discussions around long-term preventive medications, integrating patients’ life goals and preferences while providing clinicians with concrete, evidence-informed guidance. Crucially, these discussions take place within the context of the doctor–patient relationship, emphasizing that discontinuing a drug does not equate to abandoning the patient.

### Translating theory into practice: deprescribing tools and implementation

Building on our discussion of the theoretical and ethical foundations of SDM, current literature highlights how these challenges arise in practice, particularly in the context of deprescribing tools. Many existing tools provide limited information on their development and offer insufficient support for implementation in routine care [9, 13]. This underscores the importance of considering implementation from the outset.

In the development of MediQuit, key feasibility and implementation questions—such as usability in routine consultations, integration into existing workflows, and potential barriers and facilitators—were therefore addressed early and iteratively. Stakeholder input played a central role in shaping core design elements, including the three-category structure and the linear model for preventive medications, both intended to support value-sensitive decision making within real-world consultations.

These findings suggest that early integration of implementation considerations and continuous stakeholder involvement can support the development of tools that are both conceptually sound and practically applicable. At the same time, it remains uncertain to what extent these design features will translate into improved usability and uptake in routine practice, which will need to be evaluated in the planned feasibility study.

Previous research has identified patient education, time and resource constraints, and the quality of the therapeutic relationship as key determinants of successful deprescribing [37]. While these aspects informed the design of MediQuit, their effective integration into routine care cannot be assumed and will depend on contextual factors such as practice organization and consultation time.

Experiences from earlier arriba tools reinforced the importance of grounding the development of complex interventions in established frameworks [17, 38]. In contrast to previous projects with limited prospective attention to implementation, the revision of MediQuit was guided by MRC principles, with iterative refinement, stakeholder involvement, and early consideration of implementation pathways. The application of IPDAS principles further supported the development of a patient-centered and transparent tool. However, the extent to which these theoretically grounded approaches will facilitate implementation in practice remains to be demonstrated.

### Strengths and limitations

This study has benefited from the involvement of multiple stakeholders which ensured that diverse perspectives were integrated throughout the iterative refinement process. The approach has allowed for continuous refinement of the tool, enhancing both usability and clinical relevance, while explicitly considering patient life goals and ethical dimensions, aspects often neglected in existing deprescribing tools.

A further strength is the explicit alignment with IPDAS. Although IPDAS was originally developed for decision aids primarily used by patients independently, arribaMediQuit is designed to facilitate SDM directly within the consultation and is mainly operated by the physician. Despite this difference, applying IPDAS criteria provided a structured framework to ensure that the tool delivers balanced, evidence-based information on benefits and risks, incorporates patient values and preferences, and supports transparent, ethically grounded SDM.

Despite these strengths, several limitations should be considered. The development and planned evaluation of arribaMediQuit are currently focused on the primary care setting, assuming that primary care physicians have a coordinating role and access to comprehensive medication information. This may not fully reflect more fragmented care situations, for example in patients primarily managed by multiple specialists.

In addition, while the tool is intended for patients with polypharmacy, its use in cases of extensive multimorbidity with a large number of medications may be limited by time constraints in routine consultations, making a selective application more feasible. The tool is also designed for use in consultations with patients who are able to express their preferences and may therefore be less applicable in patients with significant cognitive impairment or in care settings where decisions are primarily made by caregivers.

A further limitation relates to the inclusion of the factor “preference for future prevention of organ damage” in the linear model. While this concept aims to capture the importance of long-term preventive benefits, its applicability and comprehensibility may vary across different medication classes. For example, potential outcomes such as stroke or vascular complications may be more difficult to communicate in a balanced and patient-centred way, particularly without causing unnecessary concern.

## Conclusion

Effective deprescribing requires combining technical guidance with value-sensitive decision making and effective knowledge transfer.

The iterative and participatory refinement of MediQuit provides several key insights for the development and implementation of decision support tools in primary care. In particular, our findings highlight (1) the importance of clearly distinguishing between problem solving and decision making, (2) the need to explicitly address patient values in value-sensitive decisions such as deprescribing, and (3) the relevance of integrating implementation considerations early in the development process.

These insights may inform the development and refinement of similar decision support tools in primary care. The planned feasibility study will provide further insight into the tool’s real-world acceptability, usability, and implementation

## Data Availability

No quantitative or qualitative research datasets were generated or analysed during this study. The work reports on an iterative tool development process; relevant descriptive materials (e.g. design elements, development steps, figures) are included in the manuscript.
